# Comparative Efficacy of Supervised, Web-Based, and Self-Guided Exercise Interventions in Women with Patellofemoral Pain Syndrome

**DOI:** 10.3390/medicina61040731

**Published:** 2025-04-15

**Authors:** Burak Menek, Emre Dansuk

**Affiliations:** Department of Physiotherapy and Rehabilitation, Istanbul Medipol University, Beykoz 34810, Turkey; bmenek@medipol.edu.tr

**Keywords:** patellofemoral pain syndrome, telerehabilitation, supervised exercise, web-based exercise, kinesiophobia

## Abstract

*Background/Objectives*: Patellofemoral pain syndrome (PFPS) is a common musculoskeletal condition that causes anterior knee pain, often linked to increased joint stress. Rehabilitation typically includes education, strength training, and functional exercises. Recently, telerehabilitation has become a promising alternative, particularly useful in improving access to care in rural areas. This study compares the effects of supervised (SE), web-based (WBE), and self-guided (SGE) exercise programs on pain, functionality, and fear of movement (kinesiophobia) in individuals with PFPS. *Materials and Methods*: Sixty female patients with PFPS participated in this randomized controlled trial. They were randomly assigned to one of three groups: SE, WBE, or SGE. Each program lasted six weeks, with exercises adjusted based on individual tolerance. Outcomes were assessed using the Kujala Anterior Knee Pain Scale, the visual analog scale (VAS) for pain, the Timed Up and Go Test (TUG) for mobility, and the Tampa Kinesiophobia Scale. *Results*: All groups showed significant improvements in pain, functionality, and kinesiophobia (*p* < 0.05). The SE group achieved the greatest improvements across all measures, reducing pain and kinesiophobia while enhancing functionality (*p* < 0.017). The WBE group also showed significant improvements, outperforming the SGE group in all outcomes (*p* < 0.017). The SGE group demonstrated the least improvement but still achieved positive changes. *Conclusions*: Supervised exercise programs were the most effective in managing PFPS symptoms. However, the web-based programs also provided substantial benefits, making them a viable option when in-person supervision is not feasible. Future research should aim to enhance digital interventions for broader accessibility and engagement. Trial Registration: The study protocol was also registered on ClinicalTrials.gov (NCT06625086).

## 1. Introduction

Patellofemoral pain syndrome (PFPS) is a musculoskeletal disorder characterized by anterior knee pain, typically resulting from increased stress on the patellofemoral joint. It is associated with high prevalence rates in the general population [1]. Although PFPS is sometimes colloquially referred to as “chondromalacia patella”, strictly speaking, these terms are not interchangeable. Chondromalacia patella describes a pathologic softening and degeneration of the articular cartilage on the underside of the patella, as seen on imaging or arthroscopy. In contrast, PFPS is a clinical syndrome characterized by anterior knee pain with no overt structural damage required for diagnosis. Chondromalacia patella may be a possible cause (or consequence) of anterior knee pain; however, it is not synonymous with PFPS. Current perspectives do not include definitive cartilage pathologies within the definition of PFPS; therefore, conditions such as chondromalacia patella and patellar tendinopathy should be considered separately in the differential diagnosis of anterior knee pain [2]. Knee-related complaints rank as the second most common musculoskeletal condition, with patellofemoral pain recognized as one of the most prevalent subtypes. Its prevalence in the general population is reported to range between 15% and 45%. Patellofemoral pain is typically non-traumatic in origin and is characterized by diffuse anterior knee pain experienced during activities that load the joint, such as squatting, running, and ascending or descending stairs [3,4]. Patellofemoral disorders can affect individuals across various populations; however, they are most commonly observed among physically active youth and are notably more frequent in females, with prevalence rates approximately double those seen in males [3]. This increased susceptibility has been attributed to a combination of anatomical characteristics, hormonal influences, knee joint laxity, and neuromuscular imbalances, with anatomical components being the most frequently highlighted in the literature [5]. The cause of PFPS cannot be attributed to a single factor; rather, it is thought that a multifactorial etiology contributes to the development of patellofemoral issues [6]. These factors include abnormal patellar displacement; quadriceps femoris weakness; delayed activation of the vastus medialis obliquus; iliotibial band tightness; hamstring shortening; weakness of the hip extensors, abductors, and external rotators; delayed activation of the gluteus medius; and increased activity of the gluteus maximus [7,8,9,10,11]. The diagnosis of PFPS is primarily based on clinical assessment, emphasizing detailed patient history and careful physical examination. However, imaging modalities often play a supplementary role in confirming or ruling out associated structural pathologies. Among these modalities, X-ray is frequently utilized as an initial imaging method to detect potential anatomical alterations within the knee structure. Additionally, computed tomography (CT) may be employed for detailed evaluations of the patellar and trochlear anatomical features, whereas magnetic resonance imaging (MRI) is particularly beneficial due to its high soft tissue resolution and multiplanar imaging capabilities, enabling the comprehensive assessment of intra-articular structures and soft tissue conditions of the knee. Currently, the diagnosis of this condition primarily relies on a comprehensive assessment of the patient’s medical history, supported by diagnostic imaging techniques such as magnetic resonance imaging (MRI) and ultrasonography [12]. Most treatment approaches for PFPS are conservative, with surgical intervention being rarely required. Multimodal physiotherapy programs, incorporating patient education, lower extremity strengthening, flexibility, proprioception, and stretching exercises, are commonly employed to alleviate symptoms [5,13]. Targeted interventions like vastus medialis obliquus (VMO) retraining and quadriceps strengthening have been shown to reduce joint stress and improve knee function, leading to long-term pain relief [14]. Additionally, combining these exercises with weight loss, transcutaneous electrical nerve stimulation (TENS), and orthotic support can enhance rehabilitation outcomes [15]. Owing to the widespread availability of physical therapy modalities, contemporary management approaches have evolved to incorporate a variety of combined treatment methods. These include conventional biophysical agents—such as cryotherapy, ultrasound therapy, and laser therapy—as well as therapeutic exercise programs and taping techniques [16,17].

In recent years, telerehabilitation has emerged as a commonly employed approach for managing patient care remotely. Through these technologies, clinicians are able to evaluate patients and offer treatment guidance from a distance. Such services are particularly valuable for reducing healthcare inequities in rural regions where access to care is often limited. Although research is limited, studies have begun to demonstrate the clinical benefits of video-guided home exercise programs delivered via web-based platforms. Recent findings in orthopedic and musculoskeletal rehabilitation suggest that these programs can provide effective and reliable outcomes comparable to those of conventional in-person treatment approaches [18,19]. A recent meta-analysis highlighted the efficacy of real-time telerehabilitation in managing musculoskeletal disorders. The findings indicated that various tools, including phone and video conferencing applications, have shown effectiveness in delivering therapeutic interventions remotely [20]. The use of telerehabilitation as an alternative and cost-effective rehabilitation option in home care services is rapidly increasing. This rehabilitation method encompasses various services, including monitoring, intervention, supervision, education, and consultation [21]. To improve treatment outcomes and reduce overall costs, the widespread adoption of telerehabilitation techniques in clinical practice is necessary [22]. Studies in the literature on telerehabilitation have focused on the effectiveness of remote education, care, and rehabilitation programs [18,23,24]. Several studies on mobile-application-based telerehabilitation have shown that these applications improve accessibility, increase satisfaction and adherence, and are as effective as face-to-face treatment programs [23,25,26,27].

To the best of our knowledge, no study has compared web-based exercise programs, self-guided exercise programs, and supervised exercise interventions in individuals with PFPS. Comparing these three interventions is essential to determine the effectiveness of different treatment methods and to develop the most patient-centered approach for managing PFPS symptoms. We believe that the findings of our study will address gaps in the existing literature and contribute to a better understanding of the potential of telerehabilitation in clinical settings. Accordingly, the aim of our study was to investigate the effectiveness of a web-based exercise program on pain, functionality, and kinesiophobia in individuals with PFPS. Our hypothesis is that the web-based exercise program will yield similar results to supervised exercise methods in individuals with PFPS.

## 2. Materials and Methods

### 2.1. Study Design

This parallel-group, 1:1:1 allocation ratio, single-blind (participant), randomized controlled study was conducted at Istanbul Medipol University Hospital. Ethical approval for this research was secured from the Non-Interventional Ethics Committee at Istanbul Medipol University (Approval Number: E-10840098-772.02-6172). Both written and verbal forms of informed consent were acquired from every participant, and the study followed the ethical guidelines outlined in the Declaration of Helsinki. The study protocol was also registered on ClinicalTrials.gov (NCT06625086). This randomized controlled trial was reported in accordance with the CONSORT (Consolidated Standards of Reporting Trials) guidelines. To ensure comprehensive and transparent reporting of the intervention, its components were described using the TidiERT scale (Template for Intervention Description and Replication) checklist (Supplementary File S1).

### 2.2. Participants

Participants with PFPS, diagnosed by either an orthopedic surgeon or a physiatrist specializing in knee pain, were recruited for this randomized clinical trial. The diagnostic process was based on a detailed medical history, clinical symptoms, and physical examination findings for the patients, and each patient was evaluated using magnetic resonance imaging (MRI) as an advanced imaging method. All patients underwent advanced imaging, specifically, magnetic resonance imaging (MRI), to exclude other intra-articular pathologies and to support clinical diagnosis. Knee X-rays were not routinely performed on all patients unless specifically indicated by clinical suspicion of structural or degenerative pathology. Informed consent was obtained from all participants prior to inclusion. The inclusion of only female participants in this study is due to the higher prevalence of PFPS among women. Women are known to possess distinct biomechanical and anatomical characteristics, such as a greater Q-angle and increased dynamic knee valgus, which contribute to the development of PFPS. Additionally, hormonal effects, particularly those associated with fluctuations in estrogen levels, have been linked to changes in musculoskeletal function, prompting the focus of this study on this specific population [3,28,29].

Patients were included if they had a history of atraumatic knee pain persisting for at least three months and demonstrated hallmark signs of PFPS, such as retropatellar pain, the movie sign, or a positive patellar grind test. Additionally, participants needed to have one or more patellofemoral pain triggers, including pain during prolonged sitting, squatting, kneeling, or while descending or ascending stairs. The exclusion criteria consisted of a history of patellofemoral dislocation, subluxation, or osteoarthritis; previous knee surgery; or congenital deformities. Patients with neurological or rheumatological diseases; those who had previously undergone physical therapy or rehabilitation for PFPS; or those with knee instability, grade 2–3 ligament tears, or meniscal tears were also excluded.

**Figure 1:**
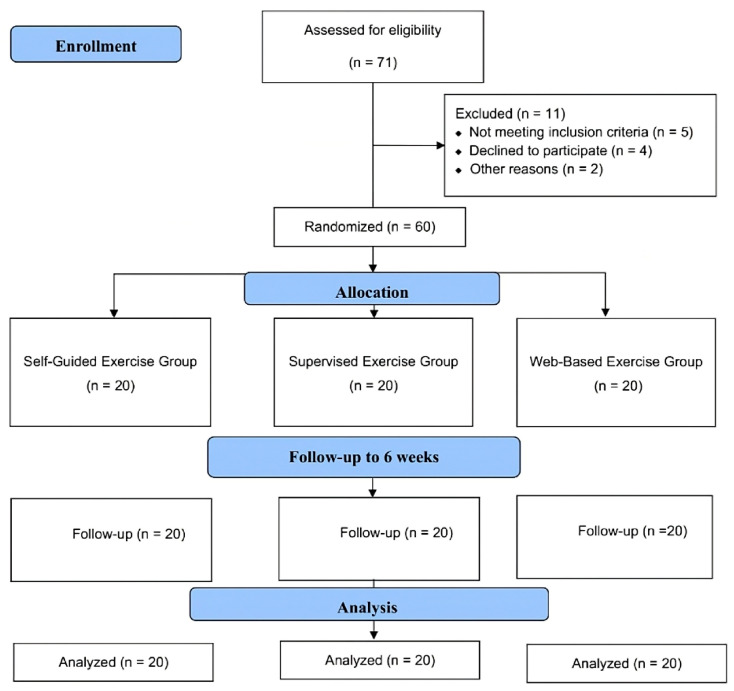
Design and flow chart of the study.

Throughout the study period, 71 patients diagnosed with PFPS were screened, of which 60 patients met the inclusion criteria. Two participants were excluded due to transportation difficulties, one was excluded due to corticosteroid treatment, one was excluded due to prior treatment at another clinic, and one was excluded due to language barriers. Four patients chose not to participate, preferring treatment from other clinics, and two others declined participation. As a result, a total of 60 eligible volunteers, with 20 patients in each group, were included in the study (Figure 1).

#### Randomization and Blinding

Numbers from 1 to 60 were randomly divided into three groups using the randomizer.org website by an independent researcher who was not involved in any part of the study, including recruitment, assessment, and intervention delivery. Participants enrolled in the study were assigned a random number according to their order of presentation at the clinic. Based on these assigned numbers, participants were allocated to the self-guided exercise (SGE), supervised exercise (SE), and web-based exercise (WBE) groups. Participants numbered 1 to 20 were assigned to the SGE group, those numbered 21 to 40 to the SE group, and those numbered 41 to 60 to the WBE group. To minimize bias, participants were blinded to their group assignments and were not informed about the interventions received by other groups. However, outcome assessors were not blinded to group allocation.

### 2.3. Interventions

#### 2.3.1. Self-Guided Exercise Group

A brochure demonstrating the treatment exercises was provided to the 20 participants in the SGE group (Supplementary Table S1). Participants were instructed to perform each exercise twice daily, 3 days a week, for 6 weeks. The exercise program was tailored to each individual’s tolerance and progression, with weekly revisions made under the supervision of a physiotherapist. Additionally, participants were given a weekly tracking sheet to record their exercises, which was reviewed by the physiotherapist each week.

#### 2.3.2. Supervised Exercise Group

Twenty participants in the SE group performed the same exercises outlined in the brochure provided to the SGE but under the direct supervision of a physiotherapist. They were instructed to perform each exercise twice daily, 3 days per week, for 6 weeks. As with the SGE group, the exercise program was tailored to each participant’s individual tolerance and progression, with adjustments made by the physiotherapist as needed.

#### 2.3.3. Web-Based Exercise Group

**Figure 2:**
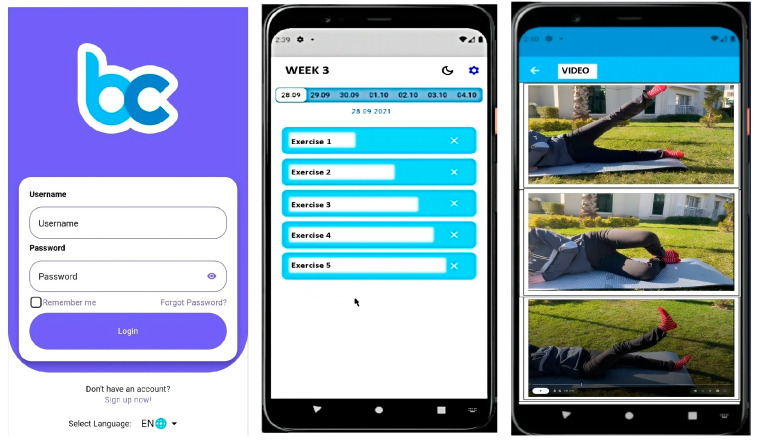
Becure mobile exercises.

The exercise program for participants in the WBE group was delivered through the Becure Mobile Application (version v.3.2.8; Becure Global, Mannheim, Germany). In collaboration with physiotherapists and engineers, the exercises outlined in the brochure were integrated into the mobile system in video format. The web-based application was developed for both the iOS and Android platforms, and each video provided detailed instructions on how to correctly perform the exercises (Figure 2). Participants were instructed, as with the other groups, to perform each exercise twice daily, three times per week, for a duration of six weeks. Additionally, a written description explaining the correct execution of each exercise was provided along with each video. Participants’ adherence to the exercise program, exercise frequency, and current health status were monitored and recorded throughout the study via the mobile application, with prompts to log their progress. Furthermore, a tracking system was implemented through the mobile application to ensure participants followed the exercises. Participants were required to confirm the completion of each exercise through the app. This method allowed us to monitor and verify whether participants consistently performed the exercises, ensuring that the implementation of and adherence to the program were effectively tracked.

### 2.4. Outcome Measures

Outcome measurements for all participants were conducted prior to the treatment and again following six weeks of treatment.

#### 2.4.1. Primary Outcomes

##### Kujala Anterior Knee Pain Scale

The primary outcome measure of our study was the Kujala score, a 13-item scale assessing knee functionality. The total Kujala score ranges from 0 to 100, with higher values indicating better performance. Additionally, the Kujala score is known for its reliability and validity in assessing patients with frozen PFPS [30].

##### Visual Analog Scale

Pain intensity was assessed using the visual analog scale. Participants were instructed to indicate their pain level on a line ranging from 0 to 10, with 0 representing no pain and 10 indicating unbearable pain [31]. Subjects marked the intensity of pain on this line according to their pain level. The severity of pain at rest and activity was recorded.

##### Timed Up and Go Test

The Timed Up and Go (TUG) test is a functional assessment used to evaluate patients’ mobility, balance, and walking abilities. Patients were instructed to start from a sitting position in a chair, stand up on command, walk as fast as possible at a safe walking speed for a predetermined distance of 3 m, turn at the endpoint, and sit on the chair. The time from the moment they stood up to the moment they sat down again was recorded with a stopwatch [32]. The test was repeated three times, and the average time was calculated.

#### 2.4.2. Secondary Outcome Measure

##### Tampa Kinesiophobia Scale

This scale consists of 17 questions that concern previous injury history or avoidance of movement due to pain. The lowest score is 17, the highest score is 68, and a score of 37 or above indicates a high level of kinesiophobia [33].

### 2.5. Sample Size

The sample size was calculated a priori using the G*Power software (version 3.0.10; Heinrich-Heine University, Düsseldorf, Germany) for a one-way ANOVA (fixed effects, omnibus, and one-way) to detect a medium effect size (f = 0.25), with an alpha level of 0.05 and statistical power (1−β) of 0.80. Based on these parameters, a minimum of 42 participants in total was required to detect statistically significant differences among the three groups. To account for potential dropout or loss to follow-up (estimated at approximately 30%), the target sample size was increased to 60 participants (20 per group). This adjustment was made to ensure sufficient statistical power would be retained despite possible attrition [34].

### 2.6. Statistical Analysis

Data analysis was performed using the SPSS software (version 20; IBM Corp., Armonk, NY, USA). The overall significance level was set at *p* < 0.05 for all analyses. The normality of the distribution of numerical variables was assessed using the Shapiro–Wilk test [35]. Intra-group and inter-group comparisons were conducted using one-way ANOVA for data following a normal distribution. The Kruskal–Wallis test was used for data that did not comply with normal distribution. To control the risk of Type I errors in multiple comparisons, the Bonferroni correction was applied, adjusting the significance threshold to *p* < 0.017. Pre- and post-treatment comparisons within groups were performed using the Wilcoxon signed-rank test for non-parametric data and the paired sample *t*-test for parametric values. Tukey’s HSD post hoc test was applied to analyze inter-group differences when ANOVA and Kruskal–Wallis results indicated significant differences. The effect size was considered “small” for 0.20–0.50, “medium” for 0.51–0.80, and “large” for 0.81 and above [36].

## 3. Results

A total of 60 individuals with PFPS were included in the study. The demographic characteristics of the participants, including age, height, weight, and duration of symptoms, were recorded, and it was determined that the three groups had similar demographic features. The demographic data of the participants are presented in Table 1. Detailed demographic characteristics including mean age, body mass index (BMI), and weight were recorded for each group. Statistical analyses showed no significant differences between the groups regarding these parameters (*p* > 0.05). Exercise adherence was systematically monitored in all groups throughout the study period. The adherence rate was 95% in the supervised group, 93% in the web-based group, and 89% in the self-guided group. Exercise adherence was similar across all three groups (*p* > 0.017). In the within-group evaluations conducted before and after treatment, significant improvements were observed in the VAS-activity, VAS-resting, Kujala, TUG, and TAMPA parameters for all three groups (Table 2, *p* < 0.05). When the differences between groups before and after treatment were examined, it was found that the SE group achieved superior results in all parameters compared to the SGE group (VAS-activity (ES = 3.30), VAS-resting (ES = 3.30), Kujala (ES = 7.72), TUG (ES = 6.37), and TAMPA (ES = 3.02), *p* < 0.017). Similarly, the WBE group also demonstrated superior outcomes in all parameters compared to the SGE group (VAS-activity (ES = 3.30), VAS-resting (ES = 3.30), Kujala (d = 5.85), TUG (ES = 4.65), and TAMPA (ES = 2.44), *p* < 0.017). In the comparison between the SE and WBE groups, both groups exhibited similar levels of improvement in the VAS-activity parameter (ES = 0.61) (*p* > 0.017). However, the SE group showed superior results compared to the WBE group in the VAS-resting (ES = 0.52), Kujala (ES = 1.33), TUG (ES = 1.97), and TAMPA (ES = 2.14) parameters (Table 3, *p* < 0.017).

## 4. Discussion

This randomized controlled trial aimed to examine the effects of both supervised and web-based telerehabilitation programs on clinical outcomes, such as pain, functionality, and kinesiophobia, in individuals with PFPS. To the best of our knowledge, no prior research has explored the impact of web-based telerehabilitation on individuals with PFPS. The findings of our study indicate that supervised exercises, web-based exercises, and exercises provided via brochures have positive effects on pain, functionality, and kinesiophobia in individuals with PFPS. However, it was found that exercises performed under physiotherapist supervision were more effective than both the web-based and brochure groups across all parameters. Additionally, web-based exercise applications demonstrated greater effectiveness than exercises provided through brochures across all parameters. Although statistically significant improvements were observed, clinical relevance was assessed by comparing the results with minimal clinically important differences (MCIDs). The supervised and web-based groups exceeded the MCID for the Kujala score, suggesting meaningful clinical improvements. Conversely, improvements in the self-guided group, despite being statistically significant, did not consistently exceed MCID thresholds, highlighting the potential limitations of unsupervised exercise regimens [37].

Consistent with previous research, PFPS prevalence is notably higher among young adults, particularly women. Epidemiological studies have demonstrated female-to-male prevalence ratios as high as 2:1, highlighting biomechanical and hormonal factors contributing to this discrepancy [3,28].

Although PFPS is fundamentally diagnosed through clinical evaluation, imaging techniques like MRI and CT can offer significant value in differential diagnosis by visualizing soft tissue and cartilage structures. MRI, due to its superior soft tissue contrast resolution, is particularly advantageous in identifying patellar cartilage lesions, effusions, or soft tissue injuries that may accompany or mimic PFPS [12].

The cornerstone of physical therapy for PFPS involves targeted strengthening exercises for quadriceps, hip abductors, and external rotators. Additional physical therapy modalities such as cryotherapy, ultrasound therapy, and laser therapy are often incorporated to manage acute pain and inflammation, despite limited standalone efficacy as highlighted by current evidence [16,17]. Moreover, Muñoz-Fernández et al. presented a case series evaluating the effects of ultrasound-guided percutaneous electrolysis combined with eccentric–concentric exercise in patients with patellar tendinopathy. Although the study design differs from that of the present research, the observed improvements in pain and function reinforce the clinical significance of multimodal, exercise-based interventions [38].

The effectiveness of telerehabilitation has been widely demonstrated across various patient groups [20,39]. Web-based telerehabilitation holds significant potential to address healthcare disparities, particularly in underserved populations. By leveraging accessible devices such as smartphones and computers, these interventions can reach individuals in rural or remote areas where access to physiotherapy services is limited. Furthermore, the reduced need for travel and in-person visits lowers financial and logistical barriers, increasing adherence to and participation in treatment programs. These features make telerehabilitation a promising tool for bridging the gap in healthcare access for vulnerable populations [40]. The distinct features of telerehabilitation provide notable advantages for each clinical population. For example, following exercises via video enhances the implementation process for patients. Additionally, the reminder function of the software offers extra motivation to adhere to the protocol [21,41]. Furthermore, clinicians can provide more comprehensive explanations by sending reminders through messages to patients. Simultaneous implementation of exercises with audio narration in videos also supports a more efficient learning process [42]. In traditional methods, patients may struggle to fully understand and correctly perform exercises due to insufficient explanations and images on paper [43]. Incorrectly performed exercises at home can hinder achieving clinical goals. The ability of patients to communicate with their clinicians and access their exercise protocols through readily available devices such as phones, tablets, or computers increases motivation, expectations, and satisfaction [44,45]. A user-friendly interface allows patients from various socio-cultural backgrounds to more easily engage in the treatment program. Video-based software, with its visual and audio feedback, makes exercises more enjoyable [42]. Monitoring progress can be difficult with paper-based exercises; however, telerehabilitation allows clinicians to adjust exercise prescriptions online, with the updated program or protocol sent directly to the patient’s inbox. These advantages of telerehabilitation may explain the improved outcomes observed in our study for web-based exercises compared to those delivered via brochures. A meta-analysis study showed that sessions supervised by a physiotherapist help individuals with knee osteoarthritis better adhere to therapeutic exercise programs, providing moderate-quality evidence of this effect [46].

Kinesiophobia is associated with function in PFPS patients and is also considered an indicator of pain and loss of function [47,48]. Evidence-based exercise interventions have been shown to reduce kinesiophobia in PFPS patients [49]. The results of our study are consistent with findings in the literature, showing a reduction in kinesiophobia across all treatment groups. Additionally, the web-based exercise intervention was found to be more effective in reducing kinesiophobia compared to exercises provided through brochures (ES: 2.44). In a study by Jin Lee et al. on individuals with PFPS, it was reported that both supervised exercise programs and telerehabilitation interventions were effective in improving kinesiophobia, pain, and functionality, but no significant superiority was found among the groups [50]. Albornoz-Cabello et al. compared patient-reported outcomes between informational brochures and physiotherapist-led telerehabilitation, finding that telerehabilitation was more effective than brochures in improving pain and functionality [51].

Chhabra et al. developed a 12-week conservative treatment program utilizing a smartphone application, incorporating medication adjustments, the promotion of physical activity, and tailored exercise guidance. Their findings indicated notable improvements in pain levels and functional impairment among patients. Additionally, telerehabilitation demonstrated greater effectiveness in enhancing functionality compared to conventional methods [52]. Another study compared online supervised exercises with home exercises in individuals with PFPS. The results indicated that online supervised exercises were more effective than home exercises in reducing pain and kinesiophobia and improving mental health parameters. These findings suggest that telerehabilitation interventions could serve as an alternative treatment approach for individuals with PFPS [53]. A meta-analysis conducted by Man et al. evaluated the effectiveness of telerehabilitation for older adults. The study’s findings highlighted that telerehabilitation interventions were more effective than traditional methods and emphasized the need for the broader use of telerehabilitation in medical rehabilitation [54]. Another study compared three groups of individuals with subacromial impingement syndrome: manual therapy, telerehabilitation, and home exercises. The findings revealed that the telerehabilitation intervention was more effective than the home exercise group in improving pain, functionality, and range of motion (ROM) parameters [55]. Another systematic review and meta-analysis demonstrated that telerehabilitation is effective in improving pain and disability in patients with chronic neck pain, with significant differences observed compared to no-intervention and control groups. The findings highlight that telerehabilitation could be a valuable alternative for patients without access to face-to-face treatment. However, further high-quality studies with long-term follow-ups are needed to confirm these findings and establish clear guidelines for the integration of telerehabilitation into clinical practice. Additionally, integrating telerehabilitation into healthcare systems could enhance accessibility and adherence, particularly for patients with limited access to traditional medical care [56]. The findings of our study are consistent with the results of these studies in the literature. Similarly, in our study, the telerehabilitation intervention demonstrated greater effectiveness compared to the exercise intervention delivered via brochures in terms of pain, functionality, and kinesiophobia parameters (VAS-activity ES: 3.30, Kujala ES: 5.85, and TAMPA ES: 2.44).

The results of our study align with the literature, showing that supervised exercise and web-based exercise interventions are more effective than brochure-based exercises in improving pain, functionality, and kinesiophobia. Unlike previous findings, our study found that the supervised exercise group was more effective than web-based interventions in reducing pain and improving functionality and kinesiophobia. We believe that telerehabilitation interventions could be an effective alternative when in-person treatment is not feasible.

This study is one of the few that have investigated the effectiveness of web-based telerehabilitation in individuals with PFPS, addressing a significant gap in the literature. By comparing supervised, web-based, and self-guided exercises, it provides a comprehensive evaluation of different rehabilitation methods. The findings emphasize that telerehabilitation offers an accessible and practical alternative for patients who lack access to face-to-face treatment. Specifically, telerehabilitation delivered via mobile applications enhances patient adherence and engagement through innovative features such as visual and auditory feedback, reminder notifications, and progress tracking. This approach has the potential to reduce healthcare disparities and provides a crucial solution for individuals in rural or remote areas. Furthermore, telerehabilitation is a cost-effective option, offering economic advantages for both individuals and healthcare systems. Supported by studies in the literature highlighting the effectiveness of telerehabilitation in musculoskeletal conditions, this method is not only applicable to PFPS but also holds promise for a variety of clinical conditions. The ability of physiotherapists to manage and adapt personalized treatment plans remotely further underscores the value of telerehabilitation in modern healthcare delivery.

Despite its strengths, this study has several limitations. First, the intervention period was relatively short, lasting only six weeks. This limited our ability to evaluate the long-term effects of the web-based and supervised interventions and left the sustainability of the observed improvements uncertain. Second, the study included only female participants, which restricts the generalizability of the findings to a broader population, particularly men and individuals with varying levels of physical activity. Third, factors such as socioeconomic status and access to technology, which could influence the feasibility and effectiveness of web-based interventions, were not assessed. Additionally, the lack of a no-intervention control group and the single-blind design of the study are also considered limitations. Future studies should aim to address these limitations by including larger and more diverse populations, extending follow-up periods, and evaluating factors that may impact the success of telerehabilitation programs.

## 5. Conclusions

This study demonstrates that both supervised and web-based exercise interventions significantly reduce pain, improve functionality, and decrease kinesiophobia in women with PFPS. While all three exercise approaches—supervised, web-based, and self-guided—offered beneficial effects, supervised exercises led to superior outcomes across all parameters. Importantly, web-based exercises delivered via a mobile application emerged as a viable alternative, showing greater efficacy in pain relief and functional improvement compared to self-guided exercises.

These findings underscore the potential of telerehabilitation, particularly web-based exercise programs, as an effective solution in clinical settings where in-person care is not feasible. However, considering the study’s limitations, including a relatively short intervention period, the absence of a no-intervention control group, and the exclusive inclusion of female participants, the results should be interpreted with caution. As gender-related biomechanical and hormonal differences may influence treatment response, the findings of this study apply only to the female population. Future studies should aim to evaluate the long-term effects of these interventions, include more diverse populations (including male participants), and investigate strategies to enhance patient adherence and engagement in digital rehabilitation platforms.

## Figures and Tables

**Table 1:** Characteristics of the participants.

Participants	SGE (*n* = 20)	SE (*n* = 20)	WBE (*n* = 20)
Age (years)	32.00 ± 2.05	33.40 ± 2.32	32.60 ± 2.45
Height (cm)	159.85 ± 1.18	162.10 ± 0.78	160.90 ± 1.11
Weight (kg)	69.05 ± 1.82	70.15 ± 1.98	70.65 ± 1.98
BMI	27.02 ± 0.71	26.70 ± 0.83	27.29 ± 0.80
Duration (month)	3.25 ± 0.80	3.20 ± 0.67	3.40 ± 0.93

SGE: self-guided exercise group; SE: supervised exercise group; WBE: web-based exercise group; BMI: body mass index.

**Table 2:** Comparison of values pre-treatment and post-treatment within the groups.

Variable	Pre-SGE (Mean ± SD) 95% CI (Lower/Upper)	Post-SGE (Mean ± SD) 95% CI (Lower/Upper)	*p*	Pre-SE (Mean ± SD) 95% CI (Lower/Upper)	Post-SE (Mean ± SD) 95% CI (Lower/Upper)	*p*	Pre-WBE (Mean ± SD) 95% CI (Lower/Upper)	Post-WBE (Mean ± SD) 95% CI (Lower/Upper)	***p***
VAS-activity	7.35 ± 0.47	5.58 ± 0.45	0.00	7.22 ± 0.21	3.40 ± 0.17	0.00	7.24 ± 0.18	3.55 ± 0.15	0.00
	(7.12/7.57)	(5.36/5.79)		(7.11/7.32)	(3.32/3.48)		(7.15/7.33)	(3.48/3.62)	
VAS-resting	5.12 ± 0.41	4.27 ± 0.37	0.00	5.27 ± 0.19	2.37 ± 0.22	0.00	5.17 ± 0.21	2.38 ± 0.23	0.00
	(4.94/5.29)	(4.10/4.43)		(5.18/5.35)	(2.27/2.46)		(5.07/5.26)	(2.27/2.48)	
Kujala	64.25 ± 1.44	66.75 ± 1.61	0.00	62.35 ± 1.34	79.00 ± 1.07	0.00	63.70 ± 1.12	78.05 ± 1.63	0.00
	(63.57/64.92)	(65.99/67.50)		(61.71/62.98)	(78.49/79.50)		(63.17/64.22)	(77.28/78.81)	
TUG	11.28 ± 0.17	9.22 ± 0.25	0.00	10.89 ± 0.23	7.11 ± 0.15	0.00	10.74 ± 0.17	7.45 ± 0.24	0.00
	(11.20/11.37)	(9.10/9.34)		(10.78/11.00)	(7.04/7.18)		(10.65/10.82)	(7.33/7.56)	
TAMPA	41.08 ± 0.50	39.27 ± 0.43	0.00	42.20 ± 0.40	30.38 ± 0.61	0.00	42.75 ± 0.42	32.23 ± 0.38	0.00
	(40.84/41.32)	(39.06/39.47)		(42.01/42.39)	(30.10/30.67)		(42.55/42.95)	(32.05/32.42)	

VAS: visual analog scale; TUG: Timed Up and Go; SGE: self-guided exercise group; SE: supervised exercise group; WBE: web-based exercise group; SD: standard deviation. Wilcoxon test for VAS-activity/resting and TAMPA. Paired sample *t*-test for Kujala and TUG.

**Table 3:** Intra-group differences in values pre-treatment and post-treatment and comparison of differences between groups.

Variable	SGE (Mean ± SD) 95% CI (Lower/Upper)	SE (Mean ± SD) 95% CI (Lower/Upper)	WBE (Mean ± SD) 95% CI (Lower/Upper)	Diff *p*	*p* (SGE-SE)	ES Cohen’s d	*p* (SGE-WBE)	ES Cohen’s d	*p* (SE-WBE)	ES Cohen’s d
VAS-activity	1.77 ± 0.20 (1.68–1.86)	3.81 ± 0.24 (3.70–3.92)	3.69 ± 0.25 (3.57–3.81)	0.00	0.00	3.30	0.00	3.30	0.22	0.61
VAS-resting	0.85 ± 0.27 (0.72–0.98)	2.89 ± 0.23 (2.78–3.00)	2.78 ± 0.24 (2.67–2.89)	0.00	0.00	3.30	0.00	3.30	0.00	0.52
Kujala	2.50 ± 2.11 (1.51–3.49)	16.65 ± 1.49 (15.95–17.35)	14.35 ± 1.92 (13.45–15.25)	0.00	0.00	7.72	0.00	5.85	0.00	1.33
TUG	2.06 ± 0.28 (1.93–2.19)	3.78 ± 0.25 (3.66–3.90)	3.29 ± 0.24 (3.18–3.40)	0.00	0.00	6.37	0.00	4.65	0.00	1.97
TAMPA	1.81 ± 0.58 (1.54–2.08)	11.81 ± 0.69 (11.49–12.13)	10.51 ± 0.53 (10.26–10.76)	0.00	0.00	3.02	0.00	2.44	0.00	2.14

VAS: visual analog scale; TUG: Timed Up and Go; SGE: self-guided exercise group; SE: supervised exercise group; WBE: web-based exercise group; SD: standard deviation; ES: effect size. Kruskal–Wallis test for VAS-activity/resting and TAMPA. One-way ANOVA test for Kujala and TUG.

## Data Availability

The dataset analyzed during this study is not publicly available according to the study protocol. However, de-identified data may be obtained from the corresponding author with permission from Istanbul Medipol University upon reasonable request.

## Supplementary Materials

The following supporting information can be downloaded at https://www.mdpi.com/article/10.3390/medicina61040731/s1: Supplementary Table S1: Therapeutic Exercise Program—Brochure Guide. Supplementary File S1: CONSORT and TidiERT Scale.
