# Supplementary material for: Comparative Efficacy of Supervised, Web-Based, and Self-Guided Exercise Interventions in Women with Patellofemoral Pain Syndrome
Source: Medicina (Kaunas). 2025 Apr 15;61(4):731. doi: 10.3390/medicina61040731 (PMC12029018; doi:10.3390/medicina61040731)
Supplement: Supplementary file 1 [file medicina-61-00731-s001.zip › Supplementary Table 1.pdf]

# Traditional Hip-Knee Therapeutic Exercise Program - Brochure Guide

**Information and Patient Education**

Participants were instructed not to engage in any additional strengthening exercises for sports or the lower extremities during the exercise intervention and were asked not to consult a physician or physiotherapist for their knee pain. They were advised to continue physical activity under the following conditions:
(a) if their pain during activity did not exceed 30 mm on a 100 mm Visual Analog Scale (VAS),
(b) if their knee pain did not last longer than the physical activity, and
(c) if there was no significant increase in symptoms following the activity. Participants were also instructed to record any use of analgesic or anti-inflammatory medications.

- At least 1 day of rest between sessions,
- 2 seconds of rest between repetitions, and 30 seconds of rest between sets.

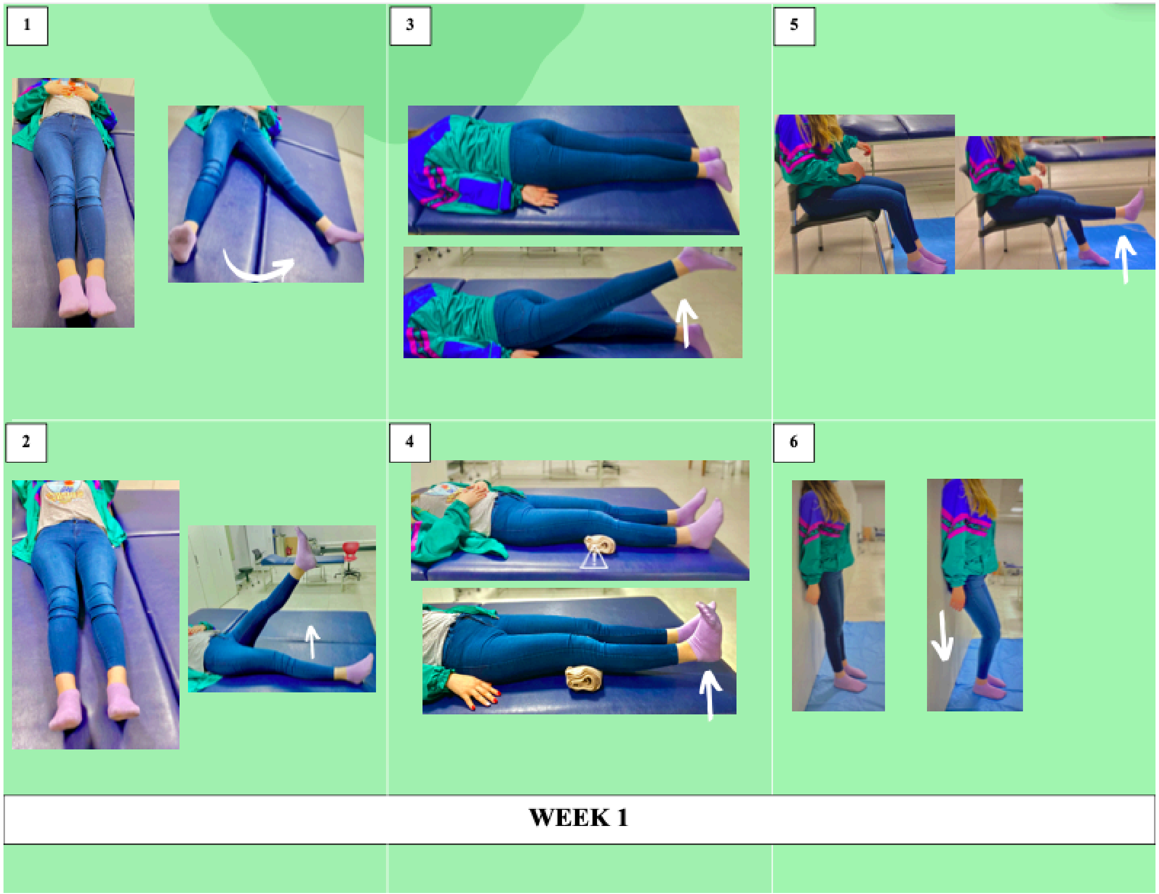


### 1. Hip Abduction in Supine Position

Lie on your back, keeping one leg stationary. Move the selected leg outward in a horizontal direction as far as possible.
Frequency: 3 days | Sets: 3 | Repetitions: 6

### 2. Hip Extension in Prone Position

Lie on your stomach. Keeping one leg stationary, lift the selected leg upwards.
Frequency: 3 days | Sets: 3 | Repetitions: 6

### 3. Knee Extension in Seated Position

Sit upright on a chair. Lift the selected leg upwards until it forms a 90-degree angle.
Frequency: 3 days | Sets: 3 | Repetitions: 6

### 4. Straight Leg Raise in Supine Position

Lie on your back. Pull your toes towards you and lift the leg upwards without bending the knee.
Frequency: 3 days | Sets: 3 | Repetitions: 6

### 5. Heel Lift in Supine Position with Rolled Towel

Lie on your back with a rolled towel under your knee. Pull your toes towards you and lift your heel without bending the knee.
Frequency: 3 days | Sets: 3 | Repetitions: 6

### 6. Wall Squat

Lean against a wall. Squat down without letting your knees pass your toes.
Frequency: 3 days | Sets: 3 | Repetitions: 6

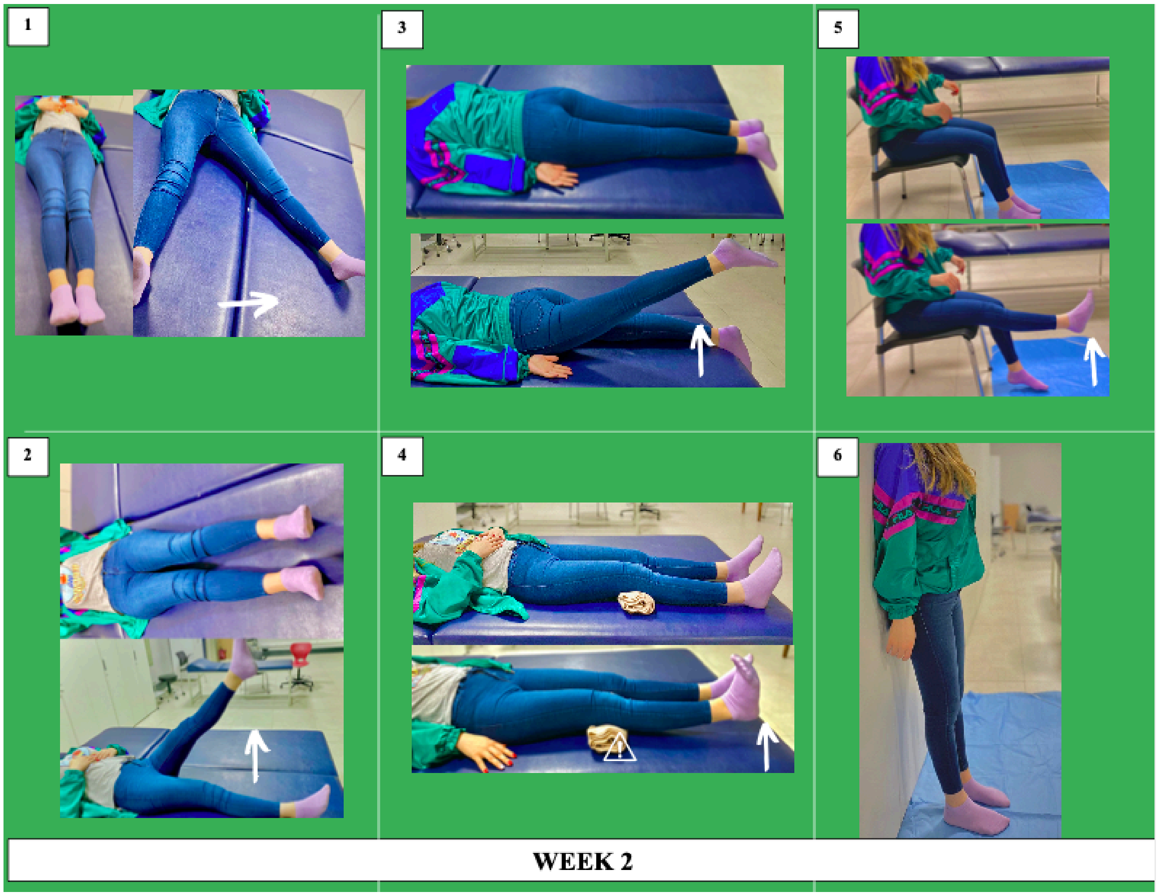


### 1. Hip Abduction in Supine Position
Lie on your back, keeping one leg stationary. Move the selected leg outward in a horizontal direction as far as possible.
Frequency: 3 days | Sets: 3 | Repetitions: 9

### 2. Hip Extension in Prone Position
Lie on your stomach. Keeping one leg stationary, lift the selected leg upwards.
Frequency: 3 days | Sets: 3 | Repetitions: 9

### 3. Knee Extension in Seated Position
Sit upright on a chair. Lift the selected leg upwards until it forms a 90-degree angle.
Frequency: 3 days | Sets: 3 | Repetitions: 9

### 4. Straight Leg Raise in Supine Position
Lie on your back. Pull your toes towards you and lift the leg upwards without bending the knee.
Frequency: 3 days | Sets: 3 | Repetitions: 9

### 5. Heel Lift in Supine Position with Rolled Towel
Lie on your back with a rolled towel under your knee. Pull your toes towards you and lift your heel without bending the knee.
Frequency: 3 days | Sets: 3 | Repetitions: 9

### 6. Wall Squat
Lean against a wall. Squat down without letting your knees pass your toes.
Frequency: 3 days | Sets: 3 | Repetitions: 6

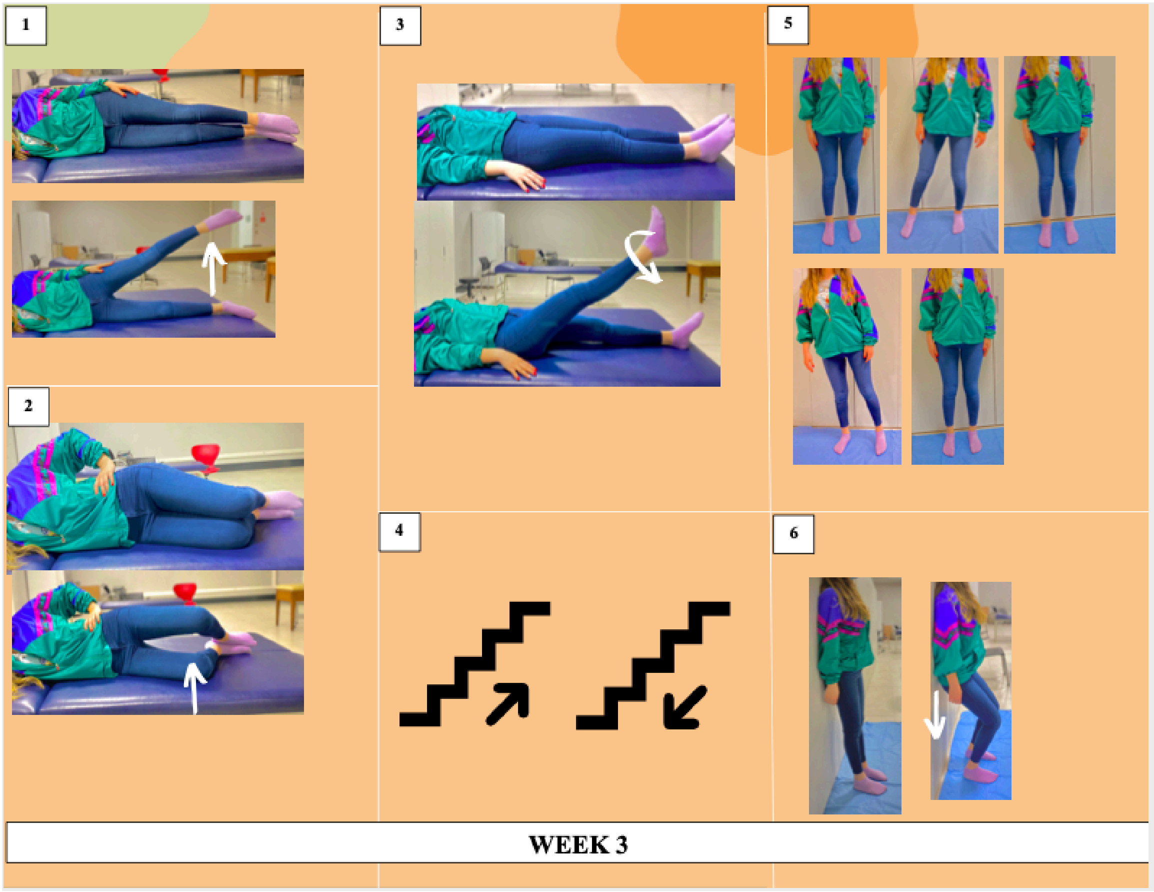


## 1. Side-Lying Hip Abduction

Lie on your side. Lift the selected leg upwards.
Frequency: 3 days | Sets: 3 | Repetitions: 6

## 2. Side-Lying Hip Clam

Lie on your side with feet together. Keep one leg stationary while lifting the other leg away.
Frequency: 3 days | Sets: 3 | Repetitions: 6

## 3. Leg Circles in Supine Position

Lie on your back. Pull your toes towards you and lift the selected leg upwards, drawing a circle with the leg.
Frequency: 3 days | Sets: 3 | Repetitions: 6

## 4. Step Climbing

Climb up and down 6 steps.
Frequency: 3 days | Sets: 3 | Repetitions: 6

## 5. Side Step

Stand upright with feet shoulder-width apart. Move the selected foot sideways, then bring the other foot to meet it. Repeat for 6 steps.
Frequency: 3 days | Sets: 3 | Repetitions: 6

## 6. Wall Squat

Lean against a wall. Squat down until your kneecaps are aligned with your toes.
Frequency: 3 days | Sets: 3 | Repetitions: 6

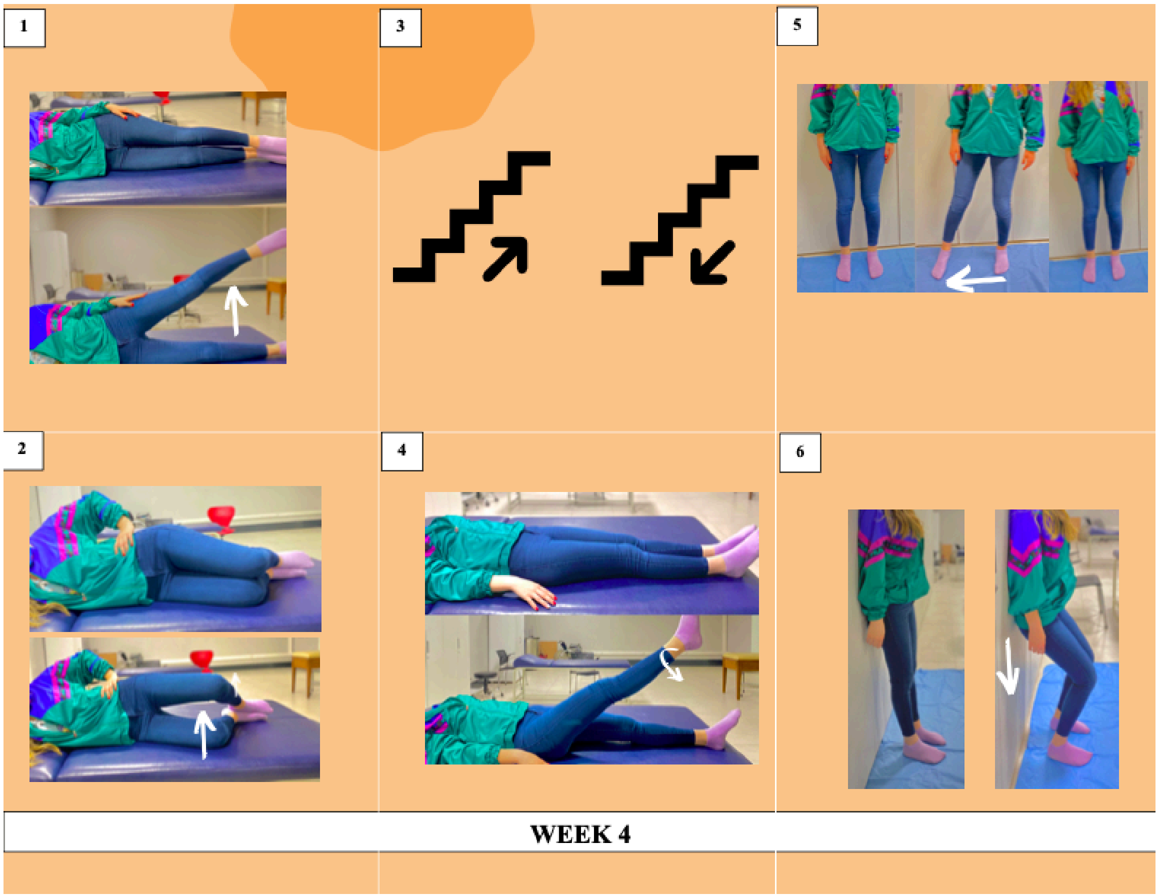


## 1. Side-Lying Hip Abduction

Lie on your side. Lift the selected leg upwards.
Frequency: 3 days | Sets: 3 | Repetitions: 9

## 2. Side-Lying Hip Clam

Lie on your side with feet together. Keep one leg stationary while lifting the other leg away.
Frequency: 3 days | Sets: 3 | Repetitions: 9

## 3. Step Climbing

Climb up and down 6 steps.
Frequency: 3 days | Sets: 3 | Repetitions: 9

## 4. Leg Circles in Supine Position

Lie on your back. Pull your toes towards you and lift the selected leg upwards, drawing a circle with the leg.
Frequency: 3 days | Sets: 3 | Repetitions: 9

## 5. Side Step

Stand upright with feet shoulder-width apart. Move the selected foot sideways, then bring the other foot to meet it. Repeat for 6 steps.
Frequency: 3 days | Sets: 3 | Repetitions: 9

## 6. Wall Squat

Lean against a wall. Squat down until your kneecaps are aligned with your toes.
Frequency: 3 days | Sets: 3 | Repetitions: 9

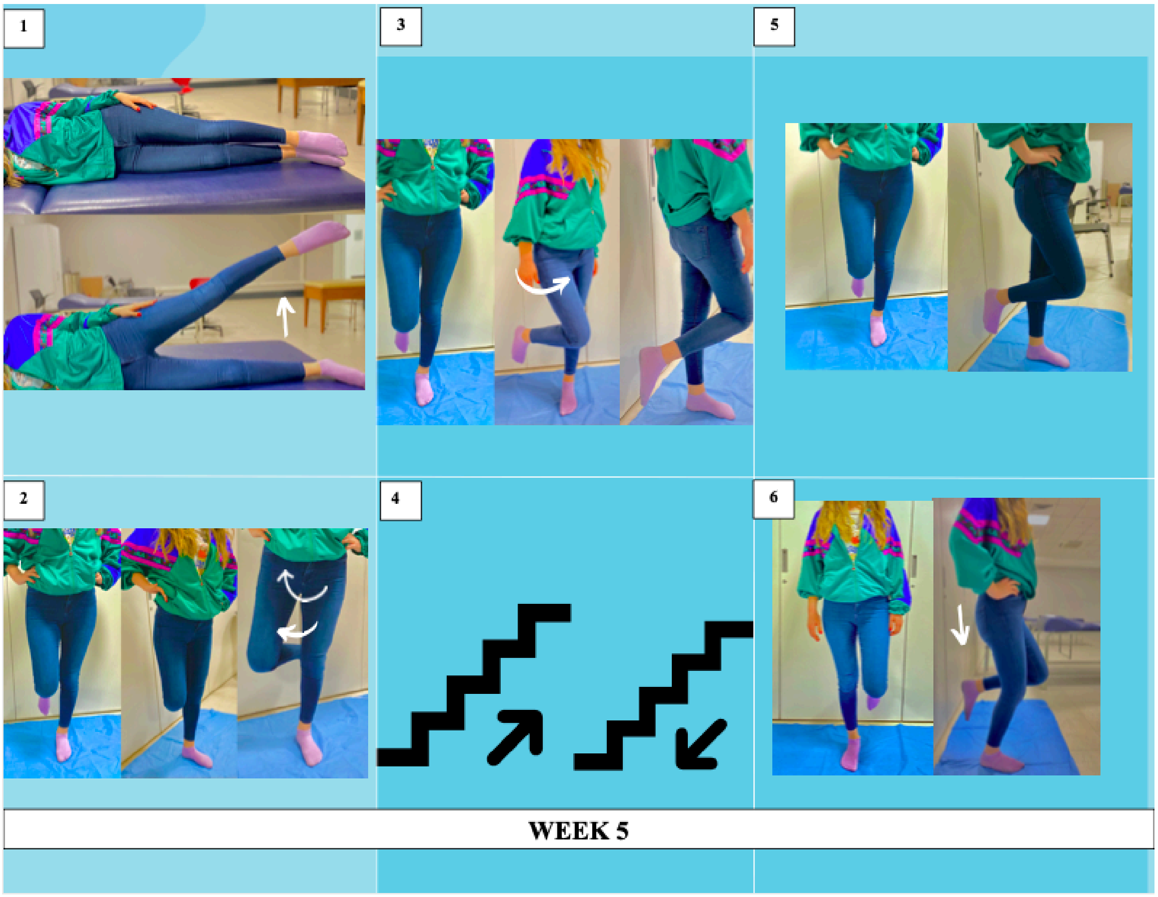


## 1. Side-Lying Hip Abduction with Hold

Lie on your side. Lift the selected leg upwards and hold for 3 seconds.
Frequency: 3 days | Sets: 3 | Repetitions: 6

## 2. Single Leg Standing with Rotation (Clockwise)

Stand on one leg. Rotate as far as possible in the direction of the elevated leg.
Frequency: 3 days | Sets: 3 | Repetitions: 6

## 3. Single Leg Standing with Rotation (Counterclockwise)

Stand on one leg. Rotate as far as possible in the opposite direction of the elevated leg.
Frequency: 3 days | Sets: 3 | Repetitions: 6

## 4. Side Step Stair Climbing

Turn sideways and climb up and down 9 steps.
Frequency: 3 days | Sets: 3 | Repetitions: 9

## 5. Single Leg Balance on Firm Surface

Stand on one leg on a firm surface for 15 seconds.
Frequency: 3 days | Sets: 3 | Repetitions: 15

## 6. Single Leg Squat

Lift one leg off the ground. Squat down without letting your knee pass your toes.
Frequency: 3 days | Sets: 3 | Repetitions: 9

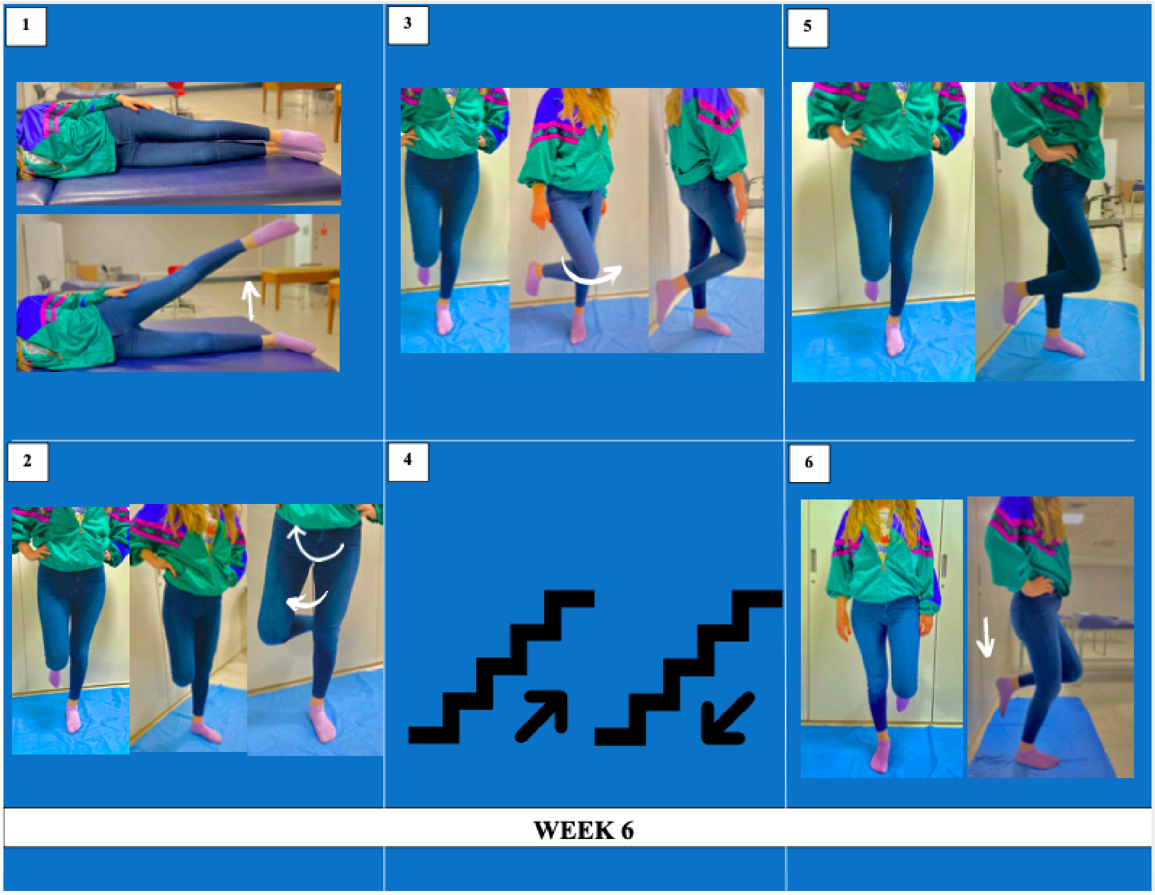


### 1. Side-Lying Hip Abduction with Hold

Lie on your side. Lift the selected leg upwards and hold for 3 seconds.
Frequency: 3 days | Sets: 3 | Repetitions: 9

### 2. Single Leg Standing with Rotation (Counterclockwise)

Stand on one leg. Rotate as far as possible in the opposite direction of the elevated leg.
Frequency: 3 days | Sets: 3 | Repetitions: 9

### 3. Single Leg Balance on Firm Surface

Stand on one leg on a firm surface for 30 seconds.
Frequency: 3 days | Sets: 3 | Repetitions: 30

### 4. Single Leg Standing with Rotation (Clockwise)

Stand on one leg. Rotate as far as possible in the direction of the elevated leg.
Frequency: 3 days | Sets: 3 | Repetitions: 9

### 5. Side Step Stair Climbing

Turn sideways and climb up and down 12 steps.
Frequency: 3 days | Sets: 3 | Repetitions: 12

### 6. Single Leg Squat

Lift one leg off the ground. Squat down without letting your knee pass your toes.
Frequency: 3 days | Sets: 3 | Repetitions: 12
